# Triglycerides as Biomarker for Predicting Systemic Lupus Erythematosus Related Kidney Injury of Negative Proteinuria

**DOI:** 10.3390/biom12070945

**Published:** 2022-07-05

**Authors:** Mingjun Si, Danyang Li, Ting Liu, Yuanyan Cai, Jingyu Yang, Lili Jiang, Haitao Yu

**Affiliations:** 1 The First Clinical Medical College, Lanzhou University, Lanzhou 730000, China; simjaaa@163.com (M.S.); lidy0908@163.com (D.L.); tliu20@lzu.edu.cn (T.L.); caiyy20@lzu.edu.cn (Y.C.); 13688496031@163.com (J.Y.); 2 School of Material Science and Technology, Lanzhou University of Technology, Lanzhou 730000, China; jianglili2002@163.com; 3 Department of Laboratory Medicine, The First Hospital of Lanzhou University, Lanzhou University, Lanzhou 730000, China

**Keywords:** systemic lupus erythematosus, association rule mining, apriori algorithm, kidney injury, triglycerides

## Abstract

Fewer biomarkers can be used to predict systemic lupus erythematosus (SLE) related kidney injury. This paper presents an apriori algorithm of association rules to mine the predictive biomarkers for SLE-related kidney injury of negative proteinuria. An apriori algorithm of association rules was employed to identify biomarkers, and logistic regression analysis and spearman correlation analysis were used to evaluate the correlation between triglycerides and SLE-related kidney injury of negative proteinuria. Triglycerides were mined out by the apriori algorithm of association rules. The level of triglycerides was significantly higher, and it was an independent risk factor for SLE-related kidney injury. In the high-triglycerides group, the number of patients with SLE-related kidney injury, SLEDAI-2K, urine P-CAST, the level of blood urea nitrogen, serum creatinine, and proteinuria were increased. Triglycerides level was positively correlated with proteinuria and P-CAST and negatively correlated with albumin and IgG. The area under the ROC curve of triglycerides and triglycerides combined proteinuria was 0.72 and 0.82, respectively. Significantly, 50% of SLE-related kidney injuries of negative proteinuria could be identified by high triglycerides levels. High triglycerides level was found at the time of onset of kidney injury, and it was opposite to glomerular filtration rate. Triglycerides may be a potential marker for predicting SLE-related kidney injury, especially in SLE-related kidney injury of negative proteinuria. Triglycerides combined proteinuria could predict SLE-related kidney injury effectively.

## 1. Introduction

Systemic lupus erythematosus (SLE) is an autoimmune disease that impairs multiple organ functions [1]. Kidney injury is a common and severe organ-specific manifestation of SLE, occurring in up to 50% of adults and 70% of children with the disease [2,3]. Kidney injury could deteriorate the prognosis of patients with SLE, and the early diagnosis and active evaluation of patients with SLE-related kidney injury play a pivotal role in promoting its therapeutic effect [4]. Conventional laboratory markers, such as urinary protein-to-creatinine ratio, complement 3, complement 4, and anti-dsDNA antibodies, are not sensitive or specific enough for predicting kidney damage in patients with SLE. As a routine marker for clinical evaluation of renal injury, proteinuria is associated with postoperative acute kidney injury (AKI) and is a valuable risk-stratification tool in the post-AKI period [5,6]. However, it plays a limited role in predicting ongoing or relapsing SLE-related kidney injury because of restrictive sensitivity, specificity, and conventional medical diagnosis methods [7,8,9]. Renal biopsy is a gold standard for diagnosing SLE-related kidney injury, and it is a useful tool for evaluating clinical efficacy [10,11]. Yet, renal biopsy is an invasive procedure with a high risk of complications, which limits its widespread application. Therefore, it is necessary to find non-invasive and effective biomarkers to improve the prediction of SLE-related kidney injury, especially in SLE-related kidney injury with negative proteinuria [7,10].

Association rule mining is a method for finding frequent co-occurrences in large databases. More recently, this method has been applied to identify clinical risk factors and analyze hidden associations with prognostic survival, which can aid in disease diagnosis and clinical management [11]. Association rule mining is based on the apriori algorithm, a popular data mining algorithm for identifying interesting correlations. In our previous report, the apriori algorithm was used to mine the biomarker of alpha-hydroxybutyrate dehydrogenase as a biomarker for predicting systemic lupus erythematosus with liver injury [12].

Triglycerides represent a major source of available lipid substrates and are the main components of lipoproteins [13,14]. Triglyceride hydrolysates include glycerol and fatty acids, which can be provided for signaling, β-oxidation, and the assembly of very-low-density lipoprotein (VLDL) [15]. Triglycerides are esters derived from glycerol and three fatty acid molecules [16]. They are hydrophobic and impossible to appear alone in blood. Under physiological conditions, triglycerides are stored in hepatocytes or exported into the blood in the form of VLDL particles. Triglyceride measurement is a direct, accurate, and precise measurement of all triglycerides in plasma, which has been applied in the diagnosis of cardiovascular diseases and hypertriglyceridemia [17]. Elevated serum triglycerides may serve as a biomarker and as causal factors for atherosclerosis and atherosclerotic cardiovascular disease [18]. It is recognized that the level of triglycerides is increased in SLE by inducing altered lipoprotein metabolism [19]. However, the value of triglycerides for predicting SLE-related kidney injury, especially kidney injury with negative proteinuria, remains unclear. 

Here, we proposed an apriori algorithm of association rule mining approach to identify an optimal logistic model of biomarkers for predicting SLE-related kidney injury of negative proteinuria. In addition, a diagnostic algorithm was proposed to predict SLE-related kidney injury.

## 2. Materials and Methods

### 2.1. Study Population

The records of 158 consecutive hospitalized cases with a diagnosis of SLE, which fulfilled the diagnostic criteria of the American College of Rheumatology in 1997, between July 2011 and January 2018, at the First Hospital of Lanzhou University were collected. The age of these patients ranged from 18 to 60 years old, 103 of them had never received immunosuppressive therapy, and the other 55 SLE patients had stopped receiving immunosuppressive therapy for more than 12 weeks. All patients who underwent blood transfusion or were diagnosed with malignancy, other autoimmune diseases, lymphoproliferative disorders, infections, and hematopoietic diseases were excluded. Additionally, 158 age- and sex-matched healthy controls were enrolled. All patients with SLE were divided into two subgroups: an SLE-related kidney injury group and an SLE-no kidney injury group. The diagnostic criteria of SLE-related kidney injury were patients with SLE who met one of the following criteria: biopsy-proven lupus nephritis (class III, IV, V, III + V, and IV + V) [20], serum creatinine > 108 μmol/L, proteinuria > 0.5 g/d, urine red blood cells > 5/HP or urine pathology cast (P-CAST)). The study protocol was approved by the Research Ethics Committee of the First Hospital of Lanzhou University (No. LDYYLL201731).

### 2.2. Laboratory Values and Clinical Assessment

Demographic data, clinical characteristics, and laboratory test results of enrolled subjects, including sex, age, body mass index, systemic lupus erythematosus disease activity index 2000 (SLEDAI-2K) score, SLE damage index, blood cell common tests, urine common tests, lactate dehydrogenase (LDH), blood urea nitrogen, serum creatinine, uric acid, the ratio of aspartate transferase to alanine transferase (AST/ALT), total cholesterol, triglycerides, high-density lipoprotein (HDL), low-density lipoprotein (LDL), albumin, total protein, α-hydroxybutyrate dehydrogenase (α-HBDH), proteinuria, complement 3, complement 4, immunoglobulin G (IgG), immunoglobulin A and immunoglobulin M, were extracted from the medical records. The estimated glomerular filtration rate (eGFR) was calculated with the indexes of serum creatinine, age, and weight. SLEDAI-2K scores greater than 4 were defined as active SLE disease.

### 2.3. Association Rule Mining (ARM) and Apriori Algorithm

ARM is a data mining technique that finds the association between an item and variables from various kinds of databases. The rule is defined as a connotation of the form A ⇒ B. The sets of items A and B are called the “antecedent” and “consequent”, respectively. Association rules are evaluated on the values of support and confidence. The support of the association rule is defined as support (%) = [number of diseases A ∩ B]/[total number of diseases], and the confidence in an association rule is defined as confidence (%) = [number of diseases A ∩ B]/[number of diseases A], where A ∩ B is the item set obtained by amalgamating A with B. The support of an item set measures its commonness, and the confidence of an association rule measures its association strength. By the essential meaning of lift, we can also define the lift for a rule, which is: lift = [(number of diseases A ∩ B) × (total number of diseases)]/[(number of diseases A) × (number of diseases B)] [21,22,23].

The process of ARM can be divided into two steps. In the first step, all the frequent itemsets that have more than minimum support in the transaction database are found. In the second step, strong association rules that meet the minimum confidence level from frequent itemsets are generated. The Apriori algorithm is a classical ARM technique, and it computes the frequent itemsets in the database through several iterations. Then, the strong association rules that meet the criteria are found from the frequent itemsets.

In our study, the itemset of association rules for 70 elements that are consisted of 54 laboratory indicators, 15 patient demographics, and 1 disease status variable. The association between the 70 elements and SLE-related kidney injury was identified by using the Apriori algorithm module in SPSS Modeler 18.0. The disease state variable was considered as an antecedent, and laboratory indicators were the consequents.

### 2.4. Statistical Analysis

The quantitative demographic data are presented as the mean ± standard deviation for normally distributed variables, and the chi-square test was used to analyze categorical variables. SPSS Modeler 18.0 was performed for data mining. Student’s *t*-test was used for normally distributed variables, and the Mann-Whitney U-test was employed for others. Logistic regression analysis was conducted to identify the independent risk factors for SLE-related kidney injury. In addition, spearman correlation analysis was used to evaluate the correlation between biomarkers and the disease activity of SLE-related kidney injury. Furthermore, a receiver operating characteristic curve (ROC) was constructed to determine the value of biomarkers for predicting SLE-related kidney injury. All data were analyzed by SPSS 22.0 statistical software (SPSS Inc., Chicago, IL, USA). *p* < 0.05 was considered statistically significant.

## 3. Results

### 3.1. Characteristics of Participants

**Figure 1:**
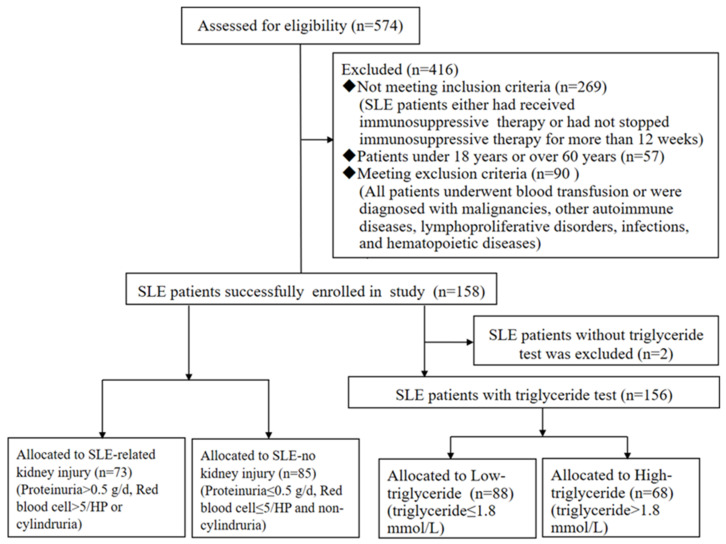
Flow diagram of the study enrolment process.

A total of 574 patients with SLE were collected, among whom 269 patients were excluded because they either had received immunosuppressive therapy or had not stopped immunosuppressive therapy for more than 12 weeks, 57 patients were excluded because their age was either less than 18 years or over 60 years, and 90 patients were excluded because they met our exclusion criteria. Thus, a total of 158 patients with SLE were enrolled in this study, and 73 (46.20%) of them had kidney injury (Figure 1). Laboratory indicators, such as triglycerides, AST/ALT, and red cell distribution width (RDW), were significantly higher in patients with SLE than in healthy controls (*p* < 0.05). However, total cholesterol, hemoglobin, platelet distribution width (PDW), hematocrit, absolute lymphocyte count (LYM), albumin, and total protein were significantly lower in patients with SLE than in healthy controls (*p* < 0.05, Table 1).

### 3.2. Data Mining

To identify laboratory indicators for diagnosing kidney injury in patients with SLE, common laboratory indicators were collected, and the correlations between common laboratory indicators and SLE with kidney injury were analyzed by data mining. According to the association rule, SLE with kidney injury was defined as the antecedent, and laboratory indicators, including routine blood examination items, blood biochemical examination items, and routine urine examination items, were defined as the consequents of the association rule. The apriori algorithm identified ten markers with strong association rules of support > 1%, confidence thresholds > 60%, and lift > 1. The results showed that triglycerides, LDH, AST/ALT, α-HBDH, total cholesterol, hemoglobin, PDW, hematocrit, RDW, and LYM were strongly associated with SLE-related kidney injury (Table 2).

### 3.3. Triglycerides May Be Independent Risk Factor for SLE-Related Kidney Injury

Patients with SLE were divided into two groups: an SLE-related kidney injury group (73/158, 46.2%) and an SLE-no kidney injury group (85/158, 53.8%). Compared with the SLE-no kidney injury group, triglycerides, LDH and α-HBDH were significantly higher, and age, hemoglobin, and hematocrit were significantly lower in SLE-related kidney injury (*p* < 0.05, Table 3).

Table 4 showed the OR of SLE-related kidney injury and triglycerides according to multiple logistic regression analysis. After adjusting for potential risk factors, such as age, gender, body mass index, hematocrit, hemoglobin, α-HBDH, and LDH, the OR (95% CI) of SLE-related kidney injury with high triglycerides was 2.44 (1.48–4.03) times than that of SLE-related kidney injury with low triglycerides. These results indicate that triglycerides may be an independent risk factor for SLE-related kidney injury.

### 3.4. Baseline Characteristics in Low- and High-Triglycerides Patients with SLE

Two SLE patients without triglycerides tests were excluded, and 156 patients with SLE were divided into two groups according to the normal upper limit of serum triglycerides (0.8–1.8 mmol/L). The level of triglycerides above the normal upper limit was classified as the high-triglycerides group, while a triglycerides level equal to or below the normal upper limit was classified as the low-triglycerides group. The demographic and clinical features and laboratory indexes were analyzed between the two groups. There were more SLE patients with kidney injury in the high-triglycerides group than that in the low-triglycerides group (69.12% vs. 29.55%; *p* < 0.001) (Table 5). The increased levels of blood urea nitrogen, creatinine, proteinuria, and P-CAST and decreased levels of IgG, albumin, and total protein could reflect kidney injury. Strikingly, SLEDAI-2K, blood urea nitrogen, creatinine, proteinuria, and P-CAST levels were significantly higher, while age, IgG, albumin, and total protein levels were significantly lower in SLE patients with high triglycerides than that with low triglycerides (*p* < 0.05, Table 5).

### 3.5. Triglycerides May Be Correlated with SLE-Related Kidney Injury

**Figure 2:**
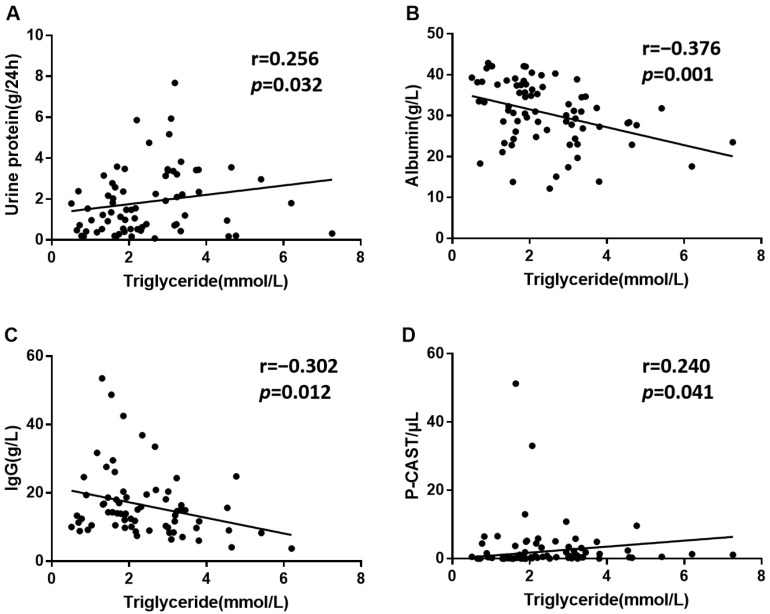
The correlations between triglycerides and indicators of SLE-related kidney injury, including urine protein (**A**), IgG (**C**), and P-CAST (**D**), were determined by Spearman correlation analysis in patients with SLE-related kidney injury. The correlation between triglycerides and albumin (**B**) was determined by Pearson correlation analysis. *p* < 0.05 was considered statistically significant. IgG: immunoglobulin G; P-CAST: urine pathology cast.

Elevated proteinuria and P-CAST and decreased serum albumin and IgG are common markers of kidney damage. Positive correlations were observed between triglycerides and proteinuria (Figure 2A) and urine P-CAST (Figure 2D), while negative correlations were shown between triglycerides and serum albumin (Figure 2B) and IgG (Figure 2C) in SLE-related kidney injury. Table S1 showed that triglycerides were associated with renal function indexes, such as kidney biopsy, urine red blood cell, and P-CAST.

### 3.6. The Predictive Value of Triglycerides for SLE-Related Kidney Injury

**Figure 3:**
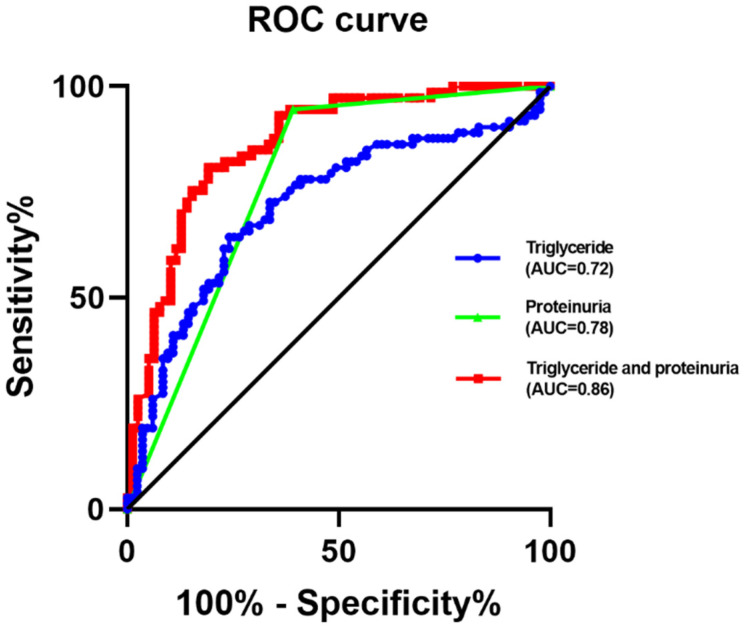
ROC curve analysis of triglycerides, proteinuria, and the combination between triglycerides and proteinuria to predict SLE-related kidney injury from SLE-no kidney injury. ROC: receiver operating characteristic.

ROC curve analysis was performed to predict the cut-off value of triglycerides to distinguish between SLE patients with and without kidney injury. The results showed that the area under the ROC curve of triglycerides was 0.72, and its optimal cut-off level was 1.84 mmol/L, which provided 64.4% sensitivity and 75.9% specificity in predicting SLE-related kidney dysfunction (Figure 3). 

Proteinuria is a key indicator for the clinical evaluation of kidney damage. The area under the ROC curve of triglycerides was 0.78. In order to promote the predictive value for SLE-related renal damage, the combination between triglycerides and proteinuria was employed. Then, the area under the ROC curve of triglycerides combined proteinuria was 0.82, which indicates an excellent value for predicting SLE-related renal damage (Figure 3).

Furthermore, proteinuria > 0.5 g/24 h was defined as positive proteinuria, while proteinuria ≤ 0.5 g/24 h was defined as negative proteinuria. Interestingly, 50% (8/16) of patients with SLE-related kidney injury with negative proteinuria could be identified by high triglycerides levels (Table 6).

### 3.7. Triglycerides May Reflect the Process of SLE-Related Kidney Injury

**Figure 4:**
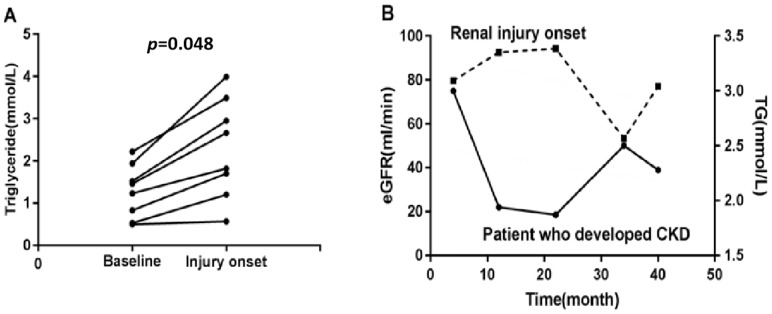
SLE patients with different levels of serum triglycerides experience different outcomes. Patients showed higher levels of triglycerides when they developed renal involvement (**A**). One patient with a persistent decreasing trend of triglycerides levels had sustained amelioration of kidney function (**B**). eGFR: estimated glomerular filtration rate; CKD: chronic kidney disease.

Eighty-five patients with SLE-no kidney injury at baseline were enrolled, and eight of them progressed to kidney injury. Among these eight SLE patients, the level of triglycerides was significantly higher at the injury onset of SLE-related kidney injury than that at baseline (*p* < 0.05, Figure 4A). Furthermore, one SLE patient had fluctuating levels of triglycerides, and the kidney injury progression was a positive correlation with the eGFR marker [19,24]. Interestingly, the level of increased triglycerides was accompanied by decreased eGFR (Figure 4B).

## 4. Discussion

Kidney injury is a common complication of SLE, while non-invasive, easily accessible, and accurate predictive markers for SLE-related renal injury are lacking [25]. The growing amount of electronically available data has augmented data sets [26]. ARM is an important data mining technique. The apriori algorithm, as a classical ARM from transaction data, is mostly deterministic and can identify the relationships between diseases and biomarkers from a large amount of data. In this study, we identified non-invasive and easily accessible biomarkers to predict SLE-related kidney injury with negative proteinuria from laboratory indicators by ARM.

Triglycerides, HDL, LDL, LDH, AST/ALT, α-HBDH, total cholesterol, hemoglobin, PDW, hematocrit, RDW, and LYM were extracted from the laboratory indicators, and all of them were significantly different between patients with SLE and healthy controls. These results are consistent with previous studies [27,28,29,30]. The nature triglycerides consist of glycerol and three fatty acids. The increased or decreased triglycerides levels are associated with human diseases [15]. The elevated triglycerides may be a result of systemic inflammation in SLE [31]. It may be caused by reduced clearance and increased synthesis of lipoproteins in SLE patients. Because HDL dysfunction in nephrotic syndrome could result in impaired LPL-mediated lipolysis of triglycerides-rich lipoproteins, this process plays a key role in dysregulating triglycerides-rich lipoprotein [32]. These observations suggest that HDL abnormalities are associated with impairment of triglycerides clearance. Patients with renal disease often have reduced clearance of triglycerides-rich lipoproteins due to hepatic lipase deficiency, which impairs the function of the liver to metabolize triglycerides [33]. In contrast to inhibiting the clearance of circulating triglycerides, inhibition of LPL by high circulating ANGPTL4 can also improve the synthesis of triglycerides [33,34]. 

We firstly demonstrated that elevated triglycerides might be an independent risk factor for SLE-related kidney injury by logistic regression analysis, and patients with SLE-related kidney injury occur mainly around 34 years of age. Furthermore, we proved that more patients with SLE had kidney injury in the high-triglycerides group than in the low-triglycerides group. These results indicate that triglycerides are correlated with SLE-related kidney injury. The mechanisms of elevated triglycerides in SLE-related kidney injury are not clear completely. The higher sugar and fat intake could result in raising triglyceride concentrations, and hyper-triglycerides may exacerbate renal damage following an inflammatory insult with increased accumulation of macrophages [35].

Proteinuria, urine P-CAST, urea nitrogen, creatinine, IgG, albumin, total protein, and SLEDAI-2K are associated with kidney damage. Our results showed that SLEDAI-2K, urea nitrogen, serum creatinine, proteinuria, and urine P-CAST levels were significantly higher, while age, IgG, albumin, and total protein levels were obviously lower in the high-triglycerides group. In addition, triglycerides were positively correlated with proteinuria and P-CAST and negatively correlated with serum albumin and IgG in patients with SLE-related kidneys. These results further illuminate that triglycerides are correlated with the disease activity of SLE-related kidney injury.

SLE-related kidney injury results in inflammatory cell infiltration, which leads to glomerular filtration barrier injury and tubular re-absorption damage in patients with SLE [36]. The reduced glomerular filtration rate causes increased serum urea nitrogen and creatinine concentrations. In our results, albumin and total protein were significantly lower in the high-triglycerides group than in the low-triglycerides group, and albumin was negatively correlated with triglycerides. The reason may be that serum albumin and total protein are filtered out through urine discharge resulting from the damaged kidneys of patients with SLE with low triglycerides.

The result of the area under the ROC curve analysis suggested that triglycerides could predict patients with SLE-related kidney injury. Proteinuria is an important biomarker for diagnosing kidney injury [37]. However, the amount of released protein is too small to detect by conventional medical diagnosis sometimes. Identifying the molecular biomarker at the early stage of the SLE-related kidney injury is the most important. Our current results showed that 50% of SLE-related kidney injury patients with negative proteinuria could be identified by high triglycerides levels. These results suggested that triglycerides may be a marker for predicting SLE-related kidney injury, especially in SLE-related kidney injury of negative proteinuria patients. Triglycerides combined with proteinuria could provide a better prediction of SLE-related kidney injury.

Our study illustrates that triglycerides level is significantly higher during the disease progression from SLE-no kidney injury to SLE-related kidney injury. A low eGFR often indicates severe kidney impairment [38]. We further found that as the level of triglycerides increased, the eGFR decreased. These results suggest that triglycerides may reflect the occurrence and progression of SLE-related kidney injury. Of course, more cases of SLE-related kidney injury should be added to further prove the association of serum triglycerides with renal function/proteinuria in the future.

## 5. Conclusions

Triglycerides may be a potential biomarker for predicting SLE-related kidney injury, especially in patients with SLE-related kidney injury of negative proteinuria. High triglycerides levels were found at the time of onset of kidney injury, and it may reflect the degree of SLE-related kidney damage. Triglycerides combined proteinuria could predict SLE-related kidney injury effectively.

## Figures and Tables

**Table 1:** Demographics and clinical characteristics of SLE patients and healthy subjects.

	SLE Patients	Healthy Subjects	*p*-Value
	(*n* = 158)	(*n* = 158)	
**Patient demographics**			
Age, years	37.05 (24.74–49.36)	36.72 (25.59–47.85)	0.953
Gender, n (%)			1.000
Male	11 (6.96)	11 (6.96)	
Female	147 (93.04)	147 (93.04)	
SLE damage index, score	0.00 (0.00–1.00)	-	-
SLEDAI-2K, score	7.00 (4.00–11.00)	-	-
BMI, kg/m^2^	21.09 (19.14–23.05)	-	-
Kidney injury, n (%)	73 (46.20)	-	-
Renal biopsy class	26 (35.62)	-	-
III/III + V	4 (15.4)	-	-
IV/IV +V	18 (69.2)	-	-
V only	4 (15.4)	-	-
Creatinine > 108 μmol/L	14 (19.18)	-	-
Proteinuria > 0.5 g/d	55 (75.34)	-	-
Red blood cell > 5/HP	50 (68.49)	-	-
P-CAST	30 (41.09)	-	-
**Laboratory indicators**			
Triglycerides, mmol/L	1.68 (1.20–2.44)	1.08 (0.81–1.45)	<0.000 ***
HDL, mmol/L	0.86 (0.65–1.11)	1.31 (1.16–1.51)	<0.000 ***
LDL, mmol/L	2.01 (1.58–2.75)	2.40 (1.69–3.11)	0.005 **
Total cholesterol, mmol/L	3.52 (2.88–4.66)	4.58 (0.82–8.34)	<0.000 ***
AST/ALT	1.53 (1.12–2.09)	1.33 (1.14–1.65)	0.003 **
Hemoglobin, g/L	108.50 (88.00–121.25)	139.19 (125.68–142.7)	<0.000 ***
PDW, fl	13.70 (12.20–16.20)	16.75 (15.93–17.57)	<0.000 ***
Hematocrit, %	32.03 (24.48–39.58)	42.85 (39.09–46.61)	<0.000 ***
RDW-SD, fl	47.50 (43.90–52.90)	42.55 (39.96–45.14)	<0.000 ***
LYM, 109/L	1.01 (0.68–1.46)	1.93 (1.44–2.42)	<0.000 ***
BUN, mmol/L	4.71 (3.51–6.83)	4.54 (3.56–5.52)	0.087
Creatinine, μmol/L	61.25 (51.00–77.28)	57.20 (52.50–65.75)	0.068
Uric acid, μmol/L	294.00 (236.00–381.25)	304.75 (227.71–381.79)	0.827
LDH, U/L	213.00 (173.00–314.75)	-	-
α-HBDH, U/L	192.00 (150.00–273.00)	-	-
Proteinuria, g/d	0.43 (0.14–1.81)	-	-
Albumin, g/L	34.21 (26.71–41.71)	46.40 (44.2–48.6)	<0.000 ***
P-CAST/μL	0.39 (0.00–1.13)	-	-
Total protein, g/L	67.14 (55.38–78.90)	77.31 (72.58–82.04)	<0.000 ***
GFR, ml/min/1.73 m^2^	128.80 (76.23–181.37)	138.45 (113.3–163.6)	0.040 *

SLE: systemic lupus erythematosus; SLEDAI-2K: systemic lupus erythematosus disease activity index 2000; BMI: body mass index; HDL: high density lipoprotein; LDL: low density lipoprotein; AST: aspartate transferase; ALT: alanine transferase; PDW: platelet distribution width; RDW-SD: red cell distribution width; LYM: absolute value of lymphocyte; BUN: blood urea nitrogen; LDH: lactic dehydrogenase; α-HBDH: α-hydroxybutyrate dehydrogenase; P-CAST: urine pathology cast; GFR: glomerular filtration rate. * *p* < 0.05, ** *p* < 0.01, *** *p* < 0.001.

**Table 2:** The laboratory indicators of connecting to SLE-related kidney injury.

Antecedent	Consequent	Lift	Support (%)	Confidence (%)
SLE-kidney dysfunction	Triglyceride	1.462	46.203	71.233
SLE-kidney dysfunction	LDH	1.341	46.203	60.274
SLE-kidney dysfunction	AST/ALT	1.176	46.203	68.493
SLE-kidney dysfunction	α-HBDH	1.173	46.203	61.644
SLE-kidney dysfunction	Total cholesterol	1.169	46.203	73.973
SLE-kidney dysfunction	Hemoglobin	1.092	46.203	78.082
SLE-kidney dysfunction	PDW	1.036	46.203	61.644
SLE-kidney dysfunction	Hematocrit	1.024	46.203	84.932
SLE-kidney dysfunction	RDW	1.006	46.203	63.014
SLE-kidney dysfunction	LYM	1.003	46.203	78.082

LDH: lactic dehydrogenase; AST: aspartatetransferase; ALT: alanine transferase; α-HBDH: α-hydroxybutyrate dehydrogenase; PDW: platelet distribution width; RDW: red cell distribution width; LYM: absolute value of lymphocyte.

**Table 3:** Univariate analysis of demographics and clinical characteristics associated with SLE-related kidney injury patients.

	SLE-Related Kidney Injury	SLE-No Kidney Injury	*p*-Value
	(*n* = 73)	(*n* = 85)	
**Patient demographics**			
Age, years	34.00 (25.00–41.50)	39.00 (28.50–46.00)	0.019 *
Gender (M/F)	5/68	6/79	0.959
Disease duration, months	12.00 (1.40–36.00)	7.00 (2.00–30.00)	0.789
BMI, kg/m^2^	21.09 (18.83–23.44)	21.00 (19.36–22.88)	0.936
**Indicators mined by association rules**			
Triglycerides, mmol/L	2.07 (1.58–3.19)	1.42 (1.07–1.82)	<0.001 ***
HDL, mmol/L	0.85 (0.60–1.11)	0.88 (0.72–1.11)	0.526
LDL, mmol/L	2.07 (1.45–2.89)	2.12 (1.33–2.91)	0.687
LDH, U/L	256.25 (187.95–350.48)	196.45 (167.40–240.00)	0.001 ***
AST/ALT	1.71 (1.10–2.58)	1.45 (1.13–1.85)	0.093
α-HBDH, U/L	231.50 (156.00–316.75)	183.00 (149.00–213.00)	0.003 **
Total cholesterol, mmol/L	3.47 (2.82–5.26)	3.53 (2.88–4.32)	0.272
Hemoglobin, g/L	100.30 (74.09–126.51)	110.06 (76.75–124.45)	0.015 *
PDW, fl	13.70 (11.60–16.05)	13.65 (12.48–16.25)	0.424
Hematocrit, %	29.95 (21.78–38.12)	34.30 (29.45–37.25)	0.001 ***
RDW-SD, fl	47.50 (44.00–52.50)	47.65 (43.83–53.45)	0.938
LYM, 109/L	0.99 (0.61–1.44)	1.05 (0.70–1.48)	0.438

BMI: body mass index; HDL: high density lipoprotein; LDL: low density lipoprotein; LDH: lactic dehydrogenase; AST: aspartatetransferase; ALT: alanine transferase; α-HBDH: α-hydroxybutyrate dehydrogenase; PDW: platelet distribution width; RDW-SD: red cell distribution width-standard deviation; LYM: absolute value of lymphocyte; * *p* < 0.05, ** *p* < 0.01, *** *p* < 0.001.

**Table 4:** Binary logistic regression analysis of risk factors associated with SLE-related kidney injury.

Index	ORs (95% CIs)	*p*-Value
Triglycerides, mmol/L	2.44 (1.48–4.03)	0.001 **
LDH, U/L	1.01 (0.10–1.03)	0.179
α-HBDH, U/L	0.99 (0.98–1.01)	0.371
Hemoglobin, g/L	1.08 (0.99–1.18)	0.074
Hematocrit, %	0.73 (0.54–1.00)	0.050
Age, years	0.99 (0.95–1.02)	0.410
Gender, male/female	0.48 (0.09–2.54)	0.388
BMI, kg/m^2^	1.02 (0.89–1.16)	0.828

LDH: lactic dehydrogenase; α-HBDH: α-hydroxybutyrate dehydrogenase; SLEDAI-2K: systemic lupus erythematosus disease activity Index 2000; BMI: body mass index; ** *p* < 0.01.

**Table 5:** Baseline characteristics of SLE patients with low and high triglycerides.

	Low-Triglycerides (*n* = 88)	High-Triglycerides (*n* = 68)	*p*-Value
**Patient demographics**			
Age, years	39.10 (27.4–50.8)	32.50 (24–45)	0.028 *
Gender, male/female	6/82	5/63	1.000
SLEDAI-2K, score	5.00 (3.25–9.00)	9.00 (4.00–14.00)	<0.001 ***
Disease duration, months	10.50 (2.00–36.00)	7.75 (2.25–24.88)	0.857
BMI	20.75 (19.23–23.18)	21.31 (18.38–24.24)	0.781
Kidney injury, n (%)	26 (29.55)	47 (69.12)	<0.001 ***
Gallbladder disorders, n (%)	19 (21.59)	13 (19.12)	0.704
Cardiovascular diseases, n (%)	16 (18.18)	20 (29.41)	0.099
Hematologic disorders, n (%)	15 (17.05)	11 (16.18)	0.885
Liver injury, n (%)	12 (13.64)	8 (11.76)	0.729
Thyroid disorders, n (%)	11 (12.50)	9 (13.24)	0.892
Splenic disorders, n (%)	7 (7.95)	4 (5.88)	0.852
Neurological symptoms, n (%)	3 (3.41)	4 (5.88)	0.726
**Laboratory indicators**			
Blood urea nitrogen, mmol/L	4.35 (3.36–5.49)	5.66 (3.79–9.29)	0.004 **
Creatinine, μmol/L	58.90 (51.00–66.15)	65.35 (51.48–98.95)	0.024 *
Anti-ds-DNA antibody (+), n (%)	17 (19.32%)	14 (20.59%)	0.888
Anti-sm antibody (+), n (%)	26 (29.55%)	23 (33.82%)	0.396
ESR, mm/h	52.00 (18.75–86.00)	43.00 (27.00–79.00)	0.644
Immunoglobulin G, g/L	17.85 (13.90–25.13)	14.90 (9.89–19.50)	0.002 **
Immunoglobulin A, g/L	2.66 (2.01–3.70)	3.01 (2.21–3.52)	0.436
Immunoglobulin M, g/L	1.38 (0.96–2.03)	1.24 (0.90–1.81)	0.650
Complement 3, g/L	0.71 (0.40–0.96)	0.50 (0.32–0.77)	0.010 *
Complement 4, g/L	0.14 (0.06–0.23)	0.095 (0.06–0.17)	0.168
CRP, mg/L	3.89 (3.27–13.05)	3.34 (3.08–9.68)	0.267
Proteinuria, g/24 h	0.25 (0.13–0.83)	0.95 (0.30–3.14)	<0.001 ***
Albumin, g/L	37.55 (31.63–42.05)	31.37 (23.93–38.81)	<0.001 ***
P-CAST/μL	0.25 (0.00–0.59)	0.52 (0.12–2.29)	0.001 **
Total protein, g/L	70.67 (60.5–80.84)	62.21 (50.22–74.2)	<0.001 ***

SLEDAI-2K: systemic lupus erythematosus disease activity index 2000; BMI: body mass index; ESR: erythrocyte sedimentation rate; CRP: c-reactive protein; P-CAST: urine pathology cast; * *p* < 0.05, ** *p* < 0.01, *** *p* < 0.001.

**Table 6:** The relationship between triglycerides and proteinuria in SLE-related kidney injury patients.

	Proteinuria (+), *n*	Proteinuria (−), *n*	Total
High triglycerides, n	37	8	45
Low triglycerides, n	17	8	25
Total	54	16	70

## Data Availability

Data will be provided upon reasonable request.

## Supplementary Materials

The following supporting information can be downloaded at: https://www.mdpi.com/article/10.3390/biom12070945/s1, Table S1. The association between triglycerides and renal function indexes in SLE-related kidney injury patients.

## References

[1] Song W., Tang D., Chen D., Zheng F., Huang S., Xu Y., Yu H., He J., Hong X., Yin L. (2020). Advances in applying of multi-omics approaches in the research of systemic lupus erythematosus. Int. Rev. Immunol..

[2] Liu Z., Davidson A. (2012). Taming lupus-a new understanding of pathogenesis is leading to clinical advances. Nat. Med..

[3] Kaplan M.J. (2011). Neutrophils in the pathogenesis and manifestations of SLE. Nat. Rev. Rheumatol..

[4] Almaani S., Meara A., Rovin B.H. (2017). Update on Lupus Nephritis. Clin. J. Am. Soc. Nephrol..

[5] Wahl T.S., Graham L.A., Morris M.S., Richman J.S., Hollis R.H., Jones C.E., Itani K.M., Wagner T.H., Mull H.J., Whittle J.C. (2018). Association Between Preoperative Proteinuria and Postoperative Acute Kidney Injury and Readmission. JAMA Surg..

[6] Hsu C.Y., Chinchilli V.M., Coca S., Devarajan P., Ghahramani N., Go A.S., Hsu R.K., Ikizler T.A., Kaufman J., Liu K.D. (2020). Post-Acute Kidney Injury Proteinuria and Subsequent Kidney Disease Progression: The Assessment, Serial Evaluation, and Subsequent Sequelae in Acute Kidney Injury (ASSESS-AKI) Study. JAMA Intern. Med..

[7] Aljaberi N., Bennett M., Brunner H.I., Devarajan P. (2019). Proteomic profiling of urine: Implications for lupus nephritis. Expert Rev. Proteom..

[8] Shidham G., Ayoub I., Birmingham D., Hebert P., Rovin B., Diamond B., Wofsy D., Hebert L. (2018). Limited Reliability of the Spot Urine Protein/Creatinine Ratio in the Longitudinal Evaluation of Patients With Lupus Nephritis. Kidney Int. Rep..

[9] Dumestre-Perard C., Clavarino G., Colliard S., Cesbron J.Y., Thielens N.M. (2018). Antibodies targeting circulating protective molecules in lupus nephritis: Interest as serological biomarkers. Autoimmun. Rev..

[10] Pacheco-Lugo L., Saenz-Garcia J., Navarro Quiroz E., Gonzalez Torres H., Fang L., Diaz-Olmos Y., Garavito de Egea G., Egea Bermejo E., Aroca Martinez G. (2019). Plasma cytokines as potential biomarkers of kidney damage in patients with systemic lupus erythematosus. Lupus.

[11] Jhang J., Tzeng I., Chou H., Jang S., Hsieh C., Ko Y., Huang H. (2020). Association Rule Mining and Prognostic Stratification of 2-Year Longevity in Octogenarians Undergoing Endovascular Therapy for Lower Extremity Arterial Disease: Observational Cohort Study. J. Med. Internet Res..

[12] Yu H., Han H., Li J., Li D., Jiang L. (2019). alpha-hydroxybutyrate dehydrogenase as a biomarker for predicting systemic lupus erythematosus with liver injury. Int. Immunopharmacol..

[13] Zickert A., Sundelin B., Svenungsson E., Gunnarsson I. (2014). Role of early repeated renal biopsies in lupus nephritis. Lupus Sci. Med..

[14] Berland C., Montalban E., Perrin E., Di Miceli M., Nakamura Y., Martinat M., Sullivan M., Davis X.S., Shenasa M.A., Martin C. (2020). Circulating Triglycerides Gate Dopamine-Associated Behaviors through DRD2-Expressing Neurons. Cell Metab..

[15] Tada H., Nohara A., Kawashiri M. (2018). Serum Triglycerides and Atherosclerotic Cardiovascular Disease: Insights from Clinical and Genetic Studies. Nutrients.

[16] Ravotti R., Worlitschek J., Pulham C., Stamatiou A. (2020). Triglycerides as Novel Phase-Change Materials: A Review and Assessment of Their Thermal Properties. Molecules.

[17] Santos-Baez L., Ginsberg H. (2020). Hypertriglyceridemia-Causes, Significance, and Approaches to Therapy. Front. Endocrinol..

[18] Ohmura H. (2019). Triglycerides as Residual Risk for Atherosclerotic Cardiovascular Disease. Circ. J..

[19] Szabo M.Z., Szodoray P., Kiss E. (2017). Dyslipidemia in systemic lupus erythematosus. Immunol. Res..

[20] Furie R., Rovin B.H., Houssiau F., Malvar A., Teng Y.K.O., Contreras G., Amoura Z., Yu X., Mok C.C., Santiago M.B. (2020). Two-Year, Randomized, Controlled Trial of Belimumab in Lupus Nephritis. N. Engl. J. Med..

[21] Qian G., Rao C.R., Sun X., Wu Y. (2016). Boosting association rule mining in large datasets via Gibbs sampling. Proc. Natl. Acad. Sci. USA.

[22] Zemedikun D.T., Gray L.J., Khunti K., Davies M.J., Dhalwani N.N. (2018). Patterns of Multimorbidity in Middle-Aged and Older Adults: An Analysis of the UK Biobank Data. Mayo Clin. Proc..

[23] Kim L., Myoung S. (2018). Comorbidity Study of Attention-deficit Hyperactivity Disorder (ADHD) in Children: Applying Association Rule Mining (ARM) to Korean National Health Insurance Data. Iran. J. Public Health.

[24] Ten Doesschate T., van Haren E., Wijma R.A., Koch B.C.P., Bonten M.J.M., van Werkhoven C.H. (2020). The effectiveness of nitrofurantoin, fosfomycin and trimethoprim for the treatment of cystitis in relation to renal function. Clin. Microbiol. Infect..

[25] Navarro-Quiroz E., Pacheco-Lugo L., Lorenzi H., Diaz-Olmos Y., Almendrales L., Rico E., Navarro R., Espana-Puccini P., Iglesias A., Egea E. (2016). High-Throughput Sequencing Reveals Circulating miRNAs as Potential Biomarkers of Kidney Damage in Patients with Systemic Lupus Erythematosus. PLoS ONE.

[26] Marx V. (2013). Biology: The big challenges of big data. Nature.

[27] Liu A.C., Yang Y., Li M.T., Jia Y., Chen S., Ye S., Zeng X.Z., Wang Z., Zhao J.X., Liu X.Y. (2018). Macrophage activation syndrome in systemic lupus erythematosus: A multicenter, case-control study in China. Clin. Rheumatol..

[28] Chen S.Y., Du J., Lu X.N., Xu J.H. (2018). Platelet distribution width as a novel indicator of disease activity in systemic lupus erythematosus. J. Res. Med. Sci..

[29] Zhou B., Xia Y.L., She J.Q. (2020). Dysregulated serum lipid profile and its correlation to disease activity in young female adults diagnosed with systemic lupus erythematosus: A cross-sectional study. Lipids Health Dis..

[30] Yang M., Ma N., Fu H.T., Wei T.T., Tang Q.Q., Qin B.D., Yang Z.X., Zhong R.Q. (2015). Hematocrit Level could Reflect Inflammatory Response and Disease Activity in Patients with Systemic Lupus Erythematosus. Clin. Lab..

[31] Reiss A.B., Jacob B., Ahmed S., Carsons S.E., DeLeon J. (2021). Understanding Accelerated Atherosclerosis in Systemic Lupus Erythematosus: Toward Better Treatment and Prevention. Inflammation.

[32] Vaziri N.D. (2016). HDL abnormalities in nephrotic syndrome and chronic kidney disease. Nat. Rev. Nephrol..

[33] Vaziri N.D. (2016). Disorders of lipid metabolism in nephrotic syndrome: Mechanisms and consequences. Kidney Int..

[34] Robciuc M.R., Naukkarinen J., Ortega-Alonso A., Tyynismaa H., Raivio T., Rissanen A., Kaprio J., Ehnholm C., Jauhiainen M., Pietilainen K.H. (2011). Serum angiopoietin-like 4 protein levels and expression in adipose tissue are inversely correlated with obesity in monozygotic twins. J. Lipid Res..

[35] Saja M., Cook H., Ruseva M., Szajna M., Pickering M., Woollard K., Botto M. (2018). A triglyceride-rich lipoprotein environment exacerbates renal injury in the accelerated nephrotoxic nephritis model. Clin. Exp. Immunol..

[36] Lv J.Q., Zhang W., Wang S., Zhao L., Ma R., Hu J.W., Wang L.J., Meng J., Zhou C.L., Lin G. (2010). The pentapeptide PLNPK inhibits systemic lupus erythematosus-associated renal damage. Inflamm. Res. Off. J. Eur. Histamine Res. Soc..

[37] Touzani S., Al-Waili N., Imtara H., Aboulghazi A., Hammas N., Falcao S., Vilas-Boas M., Arabi I.E., Al-Waili W., Lyoussi B. (2022). Arbutus Unedo Honey and Propolis Ameliorate Acute Kidney Injury, Acute Liver Injury, and Proteinuria via Hypoglycemic and Antioxidant Activity in Streptozotocin-Treated Rats. Cell Physiol. Biochem. Int. J. Exp. Cell. Physiol. Biochem. Pharmacol..

[38] Porrini E., Ruggenenti P., Luis-Lima S., Carrara F., Jimenez A., de Vries A.P.J., Torres A., Gaspari F., Remuzzi G. (2019). Estimated GFR: Time for a critical appraisal. Nat. Rev. Nephrol..

