# Supplementary material for: Triglycerides as Biomarker for Predicting Systemic Lupus Erythematosus Related Kidney Injury of Negative Proteinuria
Source: Biomolecules. 2022 Jul 5;12(7):945. doi: 10.3390/biom12070945 (PMC9312825; doi:10.3390/biom12070945)
Supplement: Supplementary file 1 [file biomolecules-12-00945-s001.zip › biomolecules-1758890-supplementary.pdf]

Supplementary Table S1**.** The association between triglycerides and renal function indexes in SLE-related kidney injury patients.

**Renal function Indexes**	**Detect (n)**	**Triglycerides**	***p* Value**
Renal biopsy	Yes (26)	2.98 (2.02–3.27)	0.004 **
	No (47)	1.88 (1.41–2.67)	
Creatinine > 108 μmol/L	Yes (14)	2.43 (1.82–3.28)	0.643
	No (59)	1.93 (1.54–3.18)	
Urine red blood cell > 5/HP	Yes (50)	2.43 (1.66–3.24)	0.037 *
	No (23)	1.78 (1.35–2.15)	
P-CAST	Yes (56)	2.33 (1.70–3.24)	0.003 **
	No (17)	1.58 (1.31–1.90)	

P-CAST: urine pathology cast; * $p < 0.05$, ** $p < 0.01$.
